# Vaccination Rates among the General Adult Population and High-Risk Groups in the United States

**DOI:** 10.1371/journal.pone.0050553

**Published:** 2012-11-30

**Authors:** Kathy Annunziata, Aaron Rak, Heather Del Buono, Marco DiBonaventura, Girishanthy Krishnarajah

**Affiliations:** 1 Health Outcomes Practice, Kantar Health, Princeton, New Jersey, United States of America; 2 Vaccine Public Policy and Advocacy, GlaxoSmithKline, Philadelphia, Pennsylvania, United States of America; 3 Global Clinical Safety and Pharmacovigilance, GlaxoSmithKline, Collegeville, Pennsylvania, United States of America; 4 Health Outcomes Practice, Kantar Health, New York, New York, United States of America; 5 US Health Outcomes and Epidemiology, Vaccines, GlaxoSmithKline, Philadelphia, Pennsylvania, United States of America; Fudan University, China

## Abstract

**Background:**

In order to adequately assess the effectiveness of vaccination in helping to control vaccine-preventable infectious disease, it is important to identify the adherence and uptake of risk-based recommendations.

**Methods:**

The current project includes data from five consecutive datasets of the National Health and Wellness Survey (NHWS): 2007 through 2011. The NHWS is an annual, Internet-based health questionnaire, administered to a nationwide sample of adults (aged 18 or older) which included items on vaccination history as well as high-risk group status. Vaccination rates and characteristics of vaccinees were reported descriptively. Logistic regressions were conducted to predict vaccination behavior from sociodemographics and risk-related variables.

**Results:**

The influenza vaccination rate for all adults 18 years and older has increased significantly from 28.0% to 36.2% from 2007 to 2011 (ps<.05). Compared with those not at high risk (25.1%), all high-risk groups were vaccinated at a higher rate, from 36.8% (pregnant women) to 69.7% (those with renal/kidney disease); however, considerable variability among high-risk groups was observed. Vaccination rates among high-risk groups for other vaccines varied considerably though all were below 50%, with the exception of immunocompromised respondents (57.5% for the hepatitis B vaccine and 52.5% for the pneumococcal vaccine) and the elderly (50.4% for the pneumococcal). Multiple risk factors were associated with increased rate of vaccination for most vaccines. Significant racial/ethnic differences with influenza, hepatitis, and herpes zoster vaccination rates were also observed (ps<.05).

**Conclusions:**

Rates of influenza vaccination have increased over time. Rates varied by high-risk status, demographics, and vaccine. There was a pattern of modest vaccination rate increases for individuals with multiple risk factors. However, there were relatively low rates of vaccination for most risk-based recommendations and all fell below national goals.

## Introduction

On an annual basis, the Advisory Committee on Immunization Practices (ACIP) reviews and updates its recommended vaccination schedule for the United States (U.S.) adult population. This schedule includes vaccination recommendations for those considered to be at high risk for certain vaccine-preventable diseases due to pre-existing health conditions or to lifestyle factors [1].

Despite these recommendations, vaccination rates have often fallen well short of targeted levels [2]–[5]. Only 21% of non-high-risk adults aged 18 and older received the influenza vaccine in 2004 and, even among high-risk groups (e.g., those over 65 years of age, healthcare workers, those with high-risk conditions including diabetes, emphysema, or coronary heart disease, among others), vaccination rates varied between 14% (pregnant women) and 70% (those over 65 years of age) [2]. Similarly, studies have found that influenza vaccination rates among those with specific chronic medical conditions including COPD, diabetes, chronic heart conditions, cancer and asthma varied between 32–56% [5]–[7]. Immunization rates of hepatitis B were also low among high-risk groups, ranging from 27%–48% [8]–[10].

Recent studies that have examined vaccination rates for high-risk groups often assess the impact of membership in a single high-risk group on the probability of vaccination [2], [4]–[7]. Data sources which include information on multiple risk groups would allow for a comparison among different high-risk groups as well as an understanding of the marginal impact of additional risk factors on the probability of vaccination. To our knowledge no study has examined the vaccination behavior of high-risk groups across multiple vaccines in a single data source, nor examined the predictors of vaccination among these high-risk groups. Based on data gathered from the 2007–2011 U.S. National Health and Wellness Survey (NHWS), we sought to address these gaps in the literature. The objective of this study was to first report the vaccination rates for the influenza, hepatitis A, hepatitis B, pneumococcal, tetanus-diphtheria (Td), tetanus, diphtheria, and pertussis (Tdap), and herpes zoster vaccines and profile the characteristics of vaccinees. The second objective was to focus on vaccines with known high-risk groups (influenza, hepatitis A, hepatitis B, pneumococcal) and report the vaccination rates and predictors of vaccination among those at high risk.

## Methods

### Ethics Statement

All respondents of the NHWS provided informed consent electronically prior to answering any survey questions. Because the survey was administered entirely online, written consent was not possible. All electronic forms of consent were saved and stored associated with each respondent’s unique identifier. All respondents were only known by a unique identifier. The survey and procedure was approved by an Institutional Review Board (Essex Institutional Review Board, Lebanon, NJ).

### Data Source

This study examines data from the NHWS. The NHWS is an annual, Internet-based health questionnaire administered to a nationwide sample of adults (aged 18 or older). The survey is fielded separately in each of the first three quarters in each year (Q1: January through March; Q2: April through June; Q3: July through September). Potential respondents for the NHWS were identified through the general panel of Lightspeed Research, a company which maintains various online respondent panels. All adults in the U.S. aged 18 and over are eligible to join this panel; respondents select into the panel by responding to advertisements in e-newsletters and online banner advertisements [11]. Members of the panel receive periodic (no more than 12 per year) invitations to participate in a variety of online surveys (e.g., consumer package goods, automotive, health, etc).

Each year, data from the Current Population Survey of the U.S. Census [12] was used to identify the relative proportions of age, gender, and racial/ethnic groups in the U.S.; these proportions were then mimicked during the recruiting of panel members (using a random stratified sampling framework) to ensure the final NHWS sample matched the demographic proportion of the U.S. (see Table S1). Additionally, Horvitz-Thompson sampling weights were calculated to allow for national projections based on NHWS data [13]. Although the methodologies do differ (see Limitations), vaccination-specific comparisons were also made between NHWS and the National Health and Information Survey (NHIS), a health-related survey that includes vaccine-related information conducted by the Centers for Disease Control and Prevention (CDC) (see Table S2).

This study includes data from five consecutive years of the NHWS: 2007 through 2011. Although the NHWS methodology, as described above, is equivalent across all five years, the overall sample size varied as follows: 63,012 (2007 NHWS), 63,000 (2008 NHWS), 75,000 (2009 NHWS), 75,000 (2010 NHWS), and 25,000 (2011 NHWS), the latter of which was only conducted during the first quarter. However, this smaller sample size and limited survey period is not believed to bias the results as comparisons of vaccination rates were made among Q1, Q2, and Q3 of 2010. The results were consistent across the three quarters with every vaccination rate in every quarter being within 0.6% of the overall vaccination rate for that year. This suggests that 2011 Q1 data will likely approximate the complete 2011 dataset despite its smaller sample size.

### Measures

#### Demographics

At each year of the survey, age, gender, race/ethnicity (non-Hispanic white, non-Hispanic black, Hispanic, Asian, or other), education (college degree or higher vs. less than a college degree), annual household income (below $25,000, $25,000 to less than $50,000, $50,000 to less than $75,000, $75,000 or more, or decline to answer), and possession of health insurance (yes vs. no) were assessed for all respondents.

#### Health history

To identify respondents based on their risk status, health history information was also collected. For the influenza vaccine, high-risk status was defined as being 65 or older, pregnant (at the time of the survey and not necessarily at the time of receiving the vaccine), immunocompromised (self-reporting a diagnosis of HIV or AIDS), diagnosed with liver disease (self-reporting a diagnosis of hepatitis B, hepatitis C, chronic liver disease, or cirrhosis), diagnosed with renal disease (self-reporting a diagnosis of either chronic kidney disease or moderate-to-severe renal disease), diagnosed with COPD (self-reporting a diagnosis of COPD, emphysema, or chronic bronchitis), diagnosed with coronary heart disease (CHD; self-reporting a diagnosis of congestive heart failure, myocardial infarction, angina, or arrhythmia), or self-reporting as alcohol dependent, diagnosed with asthma, or diagnosed with diabetes.

For the hepatitis A vaccine, high-risk status was defined as being diagnosed with liver disease (self-reporting a diagnosis of hepatitis B, hepatitis C, chronic liver disease, or cirrhosis) or a man who has sex with men (males self-identifying as bisexual or homosexual). For the hepatitis B vaccine, high-risk status was defined the same as with hepatitis A with the addition of self-reporting a diagnosis of HIV or renal disease.

For the pneumococcal vaccine, high-risk status was defined as being 65 and older, a current smoker, immunocompromised, alcohol dependent, or reporting a diagnosis of one of the following conditions: CHD, renal disease, lung conditions (self-reporting a diagnosis of COPD, emphysema, chronic bronchitis, or asthma), liver disease, or diabetes.

Although the above risk factors may not exactly align to all ACIP recommended groups, they serve as a proxy to assess risk status. Additional health history data were also measured: number of days exercised in the past month, current alcohol use (consume alcohol vs. abstain from alcohol), and quality of life (assessed using the physical [PCS] and mental component summary [MCS] scores from the Short Form 12-version 2 [14]).

#### Vaccination status

In each survey, NHWS respondents were asked if they had received the following vaccines: influenza vaccine, hepatitis A vaccine, hepatitis B vaccine, hepatitis A/B combination vaccine, pneumococcal vaccine, tetanus-diphtheria (Td), tetanus, diphtheria, and pertussis (Tdap), and herpes zoster vaccine (the latter only asked of those 60 years and older). The vaccination questions in the NHWS changed over the past five years. Until 2009, respondents were asked whether they had ever received any of the vaccines (except the influenza vaccine, which was always assessed as whether it was received in the past 12 months). Starting in 2009, respondents were asked whether they had received any of the vaccines in the past 12 months. In 2011, respondents were also asked whether they had ever received the vaccine, if they had not received them in the past 12 months (see Table 1). For the influenza vaccine only, respondents who did not receive the vaccine were asked why they did not (not important, not effective, forgot, unavailable, or other).

**Table 1:** National Health and Wellness Survey (NHWS) vaccination questions at each time point.

	NHWS Year				
	2007	2008	2009	2010	2011
**Influenza vaccine**	Have you received inthe past 12 months?	Have you received inthe past 12 months?	Have you received inthe past 12 months?	Have you received inthe past 12 months?	Have you received in the past 12 months?
**Hepatitis A vaccine**	Have you ever received?	Have you ever received?	Have you received inthe past 12 months?	Have you received inthe past 12 months?	Have you received in the past 12 months?; Have you ever received?
**Hepatitis B vaccine**	Have you ever received?	Have you ever received?	Have you received inthe past 12 months?	Have you received inthe past 12 months?	Have you received in the past 12 months?; Have you ever received?
**Hepatitis A/B combination vaccine**	N/A	Have you ever received?	Have you received inthe past 12 months?	Have you received inthe past 12 months?	Have you received in the past 12 months?; Have you ever received?
**Pneumococcal vaccine**	Have you ever received?	Have you ever received?	Have you received inthe past 12 months?	Have you received inthe past 12 months?	Have you received in the past 12 months?; Have you ever received?
**Td**	N/A	N/A	Have you received inthe past 12 months?	Have you received inthe past 12 months?	Have you received in the past 12 months?; Have you ever received?
**Tdap**	N/A	N/A	Have you received inthe past 12 months?	Have you received inthe past 12 months?	Have you received in the past 12 months?; Have you ever received?
**Herpes zoster vaccine**	N/A	N/A	Have you received inthe past 12 months?	Have you received inthe past 12 months?	N/A

N/A indicates not assessed.

As discussed above, the NHWS is conducted once per quarter for the first three quarters of each year. The item pertaining to the herpes zoster vaccine was not included in the first quarter survey in 2011 (it was included in a subsequent quarter). As a result, data on the herpes zoster vaccine was not available for this study (which only used the first quarter survey).

All patients who either reported receiving hepatitis A or a combination hepatitis A/B vaccine were considered to have gotten the hepatitis A vaccine. Similarly, all patients who either reported receiving hepatitis B or a combination hepatitis A/B vaccine were considered to have gotten the hepatitis B vaccine. The combination vaccine, in isolation, was not examined independently in the current study.

### Statistical Analysis

#### Influenza vaccination trend

Vaccination trends over time were examined by applying sampling weights and estimating the percentage of the U.S. adult population that received the influenza vaccine in each year. No other vaccine trends were analyzed because the assessment of vaccine receipt varied for all except the influenza vaccine. All pairwise statistical comparisons among these percentages were made using chi-square tests.

#### Profile of vaccinees

For brevity (since different survey years did not provide much variability), descriptive statistics of the demographics and health history of vaccinees were reported using only the most recent data available for each vaccine. In all cases, except for the herpes zoster vaccine, this was 2011. The most recent data for the herpes zoster vaccine was 2010. Data from other years are not shown. Descriptive statistics of demographic and health history variables were also reported for all vaccinees applying sampling weights to project to the population. Bivariate comparisons between vaccines were made on demographic variables of vaccinees using chi-square tests.

#### Vaccination rates among high-risk groups

To examine the effect of risk status on the vaccination rates of influenza, hepatitis A, hepatitis B, and pneumococcal vaccines, the most recent data was used (2011 in all cases). Applying sample weights, the percentages of those who were vaccinated among each high-risk group are reported. Comparisons of vaccination rates among groups of multiple risk factors (0 vs. 1 vs. 2 vs. 3–4 vs. 5+ risk factors) were made using chi-square tests. Because high-risk status may also be related to other factors that could influence vaccination behavior, logistic regression models were conducted as sensitivity analyses to predict vaccination behavior from risk status controlling for demographics (age, gender, ethnicity, education, household income, health insurance), and health history (exercise behavior, alcohol use, smoking status, physical component summary score, and mental component summary score). Risk status varied depending upon the vaccine as defined in the measures section above.

#### Predictors of vaccination among high-risk groups

To understand predictors of vaccination behavior among just those at high-risk logistic regression models were conducted to predict vaccination behavior (influenza, hepatitis A, hepatitis B, and pneumococcal vaccination only) from risk status variables controlling for demographics (age, gender, ethnicity, education, household income, health insurance), and health history (exercise behavior, alcohol use, smoking status, physical component summary score, and mental component summary score). Risk status varied depending upon the vaccine as defined in the measures section above.

All statistical comparisons were conducted at the p<.05 level. Where point estimates for vaccination rates are reported, 95% confidence intervals (CIs) of those point estimates are provided in parentheses.

## Results

### Influenza Vaccination Trend

Because a uniform question was used to evaluate the frequency of respondents reporting influenza vaccination across all five survey years, the yearly rate of vaccination could be compared. The rate of vaccination steadily increased from 28.0% (27.7%–28.5%) in the 2007 to 30.7% (30.3%–31.1%) in 2008 to 32.0% (31.6%–32.4%) in 2009 to 34.8% (34.4%–35.2%) in 2010 to 36.2% (35.6%–36.9%) in the 2011 survey, with each survey year significantly higher than the last (p<.05).

### Profile of Vaccinees

For each vaccine, vaccinees were significantly more likely to be female (see Table 2). Vaccination rates for various diseases varied by age. Respondents who received the influenza (53.4 years), pneumococcal (57.9 years), Td (47.9 years), and Tdap (46.3 years) were older than the respondents who received the hepatitis A and hepatitis B vaccines (39.2 and 40.0 years, respectively). As expected, given the CDC recommendation of vaccination of persons at least 60 years old [15], the mean age of respondents who received the herpes zoster vaccine was substantially older than for other vaccines 71.2 years. A significantly greater proportion of those who received the hepatitis A and hepatitis B vaccines were Hispanic (16.5% and 15.0%, respectively), non-Hispanic black (11.1% and 11.6%, respectively), and Asian (4.8% and 5.0%, respectively) compared with those who received the influenza, pneumococcal, Td, and Tdap vaccines (all p<.05). Conversely, those who received the herpes zoster vaccine were disproportionately non-Hispanic white (85.7%) compared to all over vaccines (all p<.05).Table 2.

**Table 2:** Demographic profile of adults (18 years and older) who received each respective vaccine.

	Influenza vaccine (past 12 months)	Hepatitis A vaccine (ever received)	Hepatitis B vaccine (ever received)	Pneumococcal vaccine (ever received)	Td vaccine (ever received)	Tdap vaccine (ever received)	Herpes zoster vaccine (ever received, 60+ only)
	82.8 M(36.2% of adults)	46.7 M(20.5% of adults)	59.9 M(26.3% of adults)	43.8 M(19.2% of adults)	96.0 M(42.1% of adults)	63.4 M(27.8% of adults)	4.2 M(7.9% of adults 60+)
Male	45.4%(44.3–46.6%)	46.7%(45.2–48.3%)	43.3%(41.9–44.7%)	39.3%(37.8–40.9%)	44.7%(43.6–45.7%)	42.3%(41.0–43.6%)	40.6%(37.8–43.5%)
Female	54.5%(53.4–55.7%)	53.3%(51.7–54.8%)	56.7%(55.3–58.1%)	60.7%(59.1–62.2%)	55.3%(54.3–56.4%)	57.7%(56.4–59.0%)	59.4%(56.5–62.3%)
Mean age (years)	53.4(52.9–53.8)	39.2(38.8–39.7)	40.0(39.5–40.4)	57.9(57.2–58.6)	47.9(47.6–48.3)	46.3(45.9–46.8)	71.2(70.7–71.6)
Age 65+	30.4%(29.3–31.6%)	7.5%(6.8–8.4%)	7.8%(7.1–8.7%)	44.3%(42.6–46.0%)	17.4%(16.5–18.3%)	15.8%(14.8–16.8%)	80.7%(78.4–82.8%)
Non-Hispanic white	73.9%(72.7–75.0%)	64.7%(63.2–66.3%)	65.8%(64.4–67.1%)	75.2%(73.5–76.9%)	73.8%(72.7–74.8%)	71.5%(70.2–72.8%)	85.7%(82.0–88.8%)
Hispanic	11.5%(10.6–12.5%)	16.5%(15.2–18.0%)	15.0%(13.8–16.2%)	10.6%(9.4–12.1%)	11.5%(10.6–12.4%)	12.4%(11.4–13.6%)	5.8%(3.7–8.8%)
Non-Hispanic black	8.9%(8.2–9.7%)	11.1%(10.2–12.2%)	11.6%(10.7–12.6%)	8.6%(7.5–9.8%)	8.8%(8.2–9.5%)	9.4%(8.6–10.3%)	3.6%(2.4–5.4%)
Asian	3.3%(3.0–3.8%)	4.8%(4.3–5.3%)	5.0%(4.5–5.5%)	2.6%(2.1–3.3%)	3.0%(2.7–3.3%)	3.8%(3.4–4.2%)	2.7%(1.4–5.3%)
Other	2.4%(2.1–2.7%)	2.8%(2.4–3.3%)	2.7%(2.3–3.2%)	2.9%(2.4–3.6%)	3.0%(2.7–3.3%)	2.9%(2.5–3.3%)	2.2%(1.1–4.2%)
Household income <$25,000	16.9%(16.0–17.9%)	19.7%(18.4–21.0%)	19.0%(17.9–20.2%)	21.5%(20.0–23.0%)	17.9%(17.0–18.7%)	17.5%(16.4–18.6%)	13.8%(11.6–16.3%)
Household income $25,000– <$50,000	27.4%(26.4–28.5%)	27.5%(26.1–29.0%)	27.3%(26.1–28.6%)	30.1%(28.5–31.6%)	27.7%(26.7–28.7%)	27.6%(26.5–28.9%)	24.2%(21.6–27.1%)
Household income over $50,000	46.9%(45.7–48.0%)	46.5%(44.9–48.0%)	47.0%(45.6–48.4%)	39.7%(38.2–41.3%)	47.5%(46.4–48.5%)	47.4%(46.1–48.7%)	51.8%(48.7–54.9%)
Decline to answer	8.9%(8.2–9.6%)	6.4%(5.7–7.1%)	6.7%(6.1–7.4%)	8.9%(7.9–9.8%)	7.0%(6.5–7.5%)	7.5%(6.9–8.2%)	10.2%(8.6–12.0%)
Have health insurance	92.2%(91.5–92.8%)	81.4%(80.0–82.6%)	82.5%(81.4–83.6%)	90.2%(89.9–91.8%)	84.3%(83.4–85.0%)	84.8%(83.8–85.8%)	98.6%97.8–99.1%)
Do not have health insurance	7.8%(7.2–8.5%)	18.6%(17.4–20.0%)	17.5%(16.4–18.6%)	9.1%(8.2–10.1%)	15.8%(15.0–16.6%)	15.2%(14.3–16.2%)	1.4%(0.9–2.2%)
Less than college education	67.6%(66.6–68.6%)	68.4%(67.1–69.7%)	67.0%(65.8–68.2%)	73.4%(72.1–74.7%)	67.3%(66.4–68.2%)	66.9%(65.8–68.0%)	52.4%(49.3–55.4%)
College education or higher	32.4%(31.4–33.4%)	31.6%(30.3–32.9%)	33.0%(31.9–34.2%)	26.6%(25.3–27.9%)	32.7%(31.8–33.6%)	33.1%(32.0–34.2%)	47.6%(44.6–50.7%)

All data is the from the 2011 NHWS dataset (N = 25,000) with the exception of the herpes zoster vaccine, which is from the 2010 NHWS dataset.

### Vaccination Rates Among High-risk Groups

**Figure 1:**
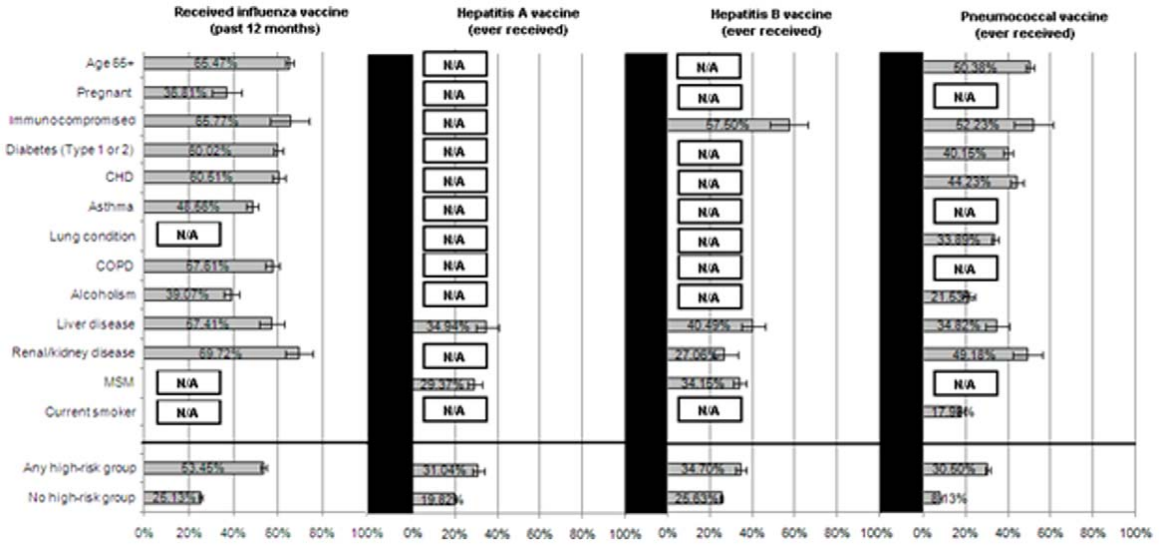
Percentage of high-risk groups who have been vaccinated. N/A: risk category is not applicable to this particular vaccine according to the ACIP; immunocompromised: (HIV/AIDS); CHD: Congestive heart failure, heart attack, angina, arrhythmia; lung conditions: COPD/emphysema/bronchitis, asthma; liver disease: Hepatitis B, Hepatitis C, chronic liver disease, cirrhosis; renal/kidney disease: chronic kidney disease, moderate-severe renal disease; MSM: men who have sex with men; Any high risk group: membership in any of the high-risk categories above.

Beginning in 2010–2011, the CDC issued a universal recommendation for the influenza vaccine for all those over 6 months of age. Prior to this recommendation, only select groups at particular risk for developing complications from influenza were universally recommended to receive the vaccine. However, the data suggests a considerable gap between the recommendation and actual vaccination rates among these high-risk groups (see Figure 1). Across all respondents classified as high-risk, only 53.5% (52.3%–54.6%) received the influenza vaccine. Between the risk groups that the survey could measure, considerable variability existed, with pregnant women (at the time of the survey) (36.8% [30.5%–43.6%]) being the least likely to have received the vaccine and those with renal/kidney disease (69.7% [63.2%–75.5%]) being the most likely. Among those who did not receive the influenza vaccine, most respondents reported that they did not do so because they believed that the vaccine was either not important (42.0% [41.1%–42.9%]) or not effective (21.2% [20.5–21.9%]).

The CDC recommends hepatitis A and B vaccination for several different high-risk groups. The NHWS captured data for a subset of those recommendations. Specifically, for those with liver disease or men who have sex with men the CDC recommends vaccination for the hepatitis.

A virus, yet only 34.9% (29.8%–40.5%) and 29.4% (26.3%–32.7%), respectively, of these high-risk respondents had ever received the vaccine (see Figure 1). Similarly, only 57.5% [48.2%–66.3%] of respondents who were immunocompromised, 40.5% [35.1%–46.1%] of those with liver disease, 27.1% [21.6%–33.4%] of those with renal/kidney disease, and 34.2% [30.9%–37.6%] among men who have sex with men (the high-risk categories for hepatitis B) had ever received the hepatitis B vaccine (see Figure 1).

Across all high-risk groups for pneumococcal infection, only 30.5% (29.5%–31.5%) had ever received the pneumococcal vaccine. Immunocompromised respondents (52.5% [43.0%–61.3%]), the elderly (50.4% [48.5%–52.3%]), and those with renal/kidney disease (49.2% [42.3%–56.1%]) were the mostly likely among the high-risk groups to vaccinate (see Figure 1). Conversely, respondents with self-identified alcohol dependency (21.5% [19.0%–24.4%]) and current smokers (18.0% [16.8%–19.3%]) were the least likely to have reported receiving pneumococcal vaccine among the high-risk groups (see Figure 1).

### Vaccination Rates Among Multiple High-risk Groups

**Figure 2:**
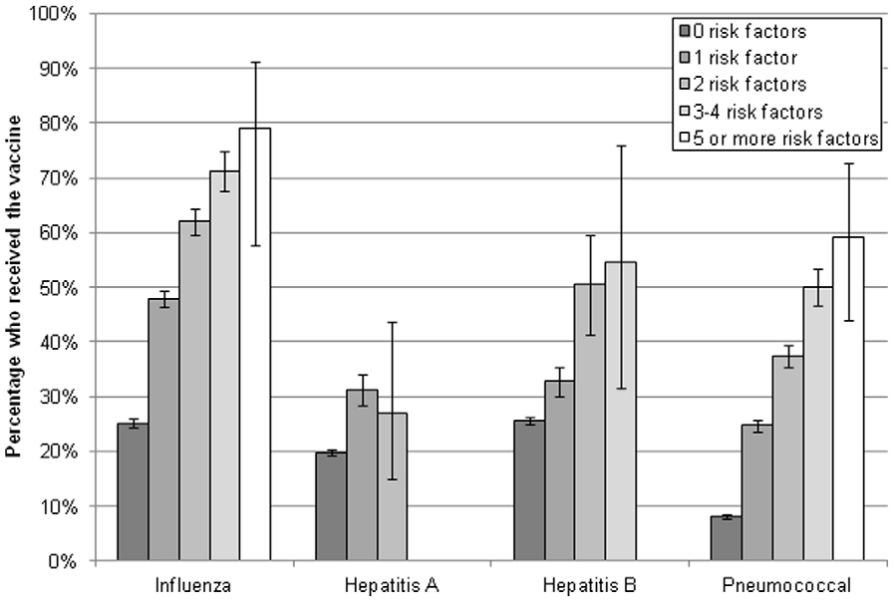
Vaccination rates by number of risk factors.

The relationship between the number of risk factors and vaccination was also examined (see Figure 2). All risk factor groups for all vaccines were significantly greater than those without risk factors (all p<.05). Significant increases in both influenza (1 risk factor: 47.8% [46.5%–49.3%], 2 risk factors: 62.1% [59.7%–64.5%], 3–4 risk factors: 71.4% [67.7%–74.8%], and 5 or more risk factors: 78.9% [57.7%–91.2%]) and pneumococcal vaccination rates (1 risk factor: 24.7% [23.6%–25.9%], 2 risk factors: 37.4% [35.3%–39.5%], 3–4 risk factors: 50.0% [46.7%–53.3%], and 5 or more risk factors: 59.2% [43.9%–72.9%]) were observed as a function of the number of risk factors, p<.05. However, for the hepatitis B vaccine, a jump in vaccination rates were observed between 1 and 2 risk factors (32.7% [30.1%–35.5%] vs. 50.5% [41.4%–59.5%]) and no difference was observed between 2 risk factors and 3–4 risk factors (50.5% [41.4%–59.5%] vs. 54.6% [31.6%–75.9%], p = .75). No difference in hepatitis A vaccination rates were observed between those with 1 risk factor (31.1% [28.4%–34.1%]) and 2 risk factors (27.1% [15.1%–43.7%]), p = .60.

Because the vaccination rate of each high-risk group was assessed independently above, a sensitivity analysis was conducted whereby each high-risk group variable was simultaneously entered into a model to predict vaccination behavior for each of these vaccines after controlling for demographic and health history variables (see Statistical Analysis section). This analysis would explain if, for example, the reason those with diabetes were receiving influenza vaccine at the rates they were was actually because of their age, rather than their diabetes. All high-risk groups for all vaccines were analyzed. However, the adjusted probabilities were nearly identical to the unadjusted probabilities reported above (data not shown).

### Predictors of Vaccination Rates Among those at High Risk

As vaccination rates among those with high-risk were generally modest, additional analyses were undertaken to better understand the predictors of vaccination for these respondents, Among those at high-risk, the strongest predictors of influenza vaccination were being immunocompromised, possession of health insurance, and the diagnosis of renal disease, diabetes, or asthma (see Table 3). Despite being risk factors, neither pregnancy nor alcohol dependency were independently associated with vaccination. Non-Hispanic blacks and those of other races were significantly less likely to receive an influenza vaccine among those at high-risk, even after controlling for risk factors and other sociodemographic factors.

**Table 3:** Predictors of vaccination among those at high-risk.

	Influenza			Hepatitis A			Hepatitis B			Pneumococcal		
	OR	95%LCL	95%UCL	OR	95%LCL	95%UCL	OR	95%LCL	95%UCL	OR	95%LCL	95%UCL
Age	1.029	1.025	1.032	0.955	0.936	0.973	0.96	0.944	0.976	1.031	1.025	1.037
Male	0.926	0.847	1.013	2.99	1.208	7.402	2.988	1.505	5.93	1.086	0.943	1.251
Non-Hispanic black	0.794	0.68	0.926	1.499	0.723	3.105	2.217	1.223	4.022	1.003	0.779	1.291
Hispanic	0.922	0.768	1.107	1.411	0.739	2.696	1.911	1.075	3.397	0.984	0.722	1.342
Asian	0.836	0.681	1.028	1.035	0.431	2.481	1.686	0.83	3.426	1.121	0.795	1.581
Other race	0.525	0.381	0.724	0.553	0.069	4.435	0.598	0.077	4.622	0.649	0.348	1.21
College educated	1.158	1.056	1.269	1.183	0.705	1.985	1.326	0.846	2.077	0.956	0.825	1.108
Household income: <$25 K	0.80	0.706	0.907	0.95	0.492	1.835	1.299	0.73	2.314	0.992	0.813	1.21
Household income: $50 K to <$75 K	1.123	0.994	1.268	0.83	0.41	1.68	1.1	0.585	2.069	1.14	0.943	1.378
Household income: $75 K and over	1.18	1.046	1.331	0.873	0.456	1.67	1.44	0.815	2.546	1.144	0.944	1.387
Household income: Decline to answer	1.062	0.91	1.239	0.678	0.191	2.401	0.651	0.188	2.253	0.949	0.737	1.221
Possess health insurance	2.415	2.085	2.798	1.406	0.763	2.589	1.365	0.783	2.382	1.783	1.355	2.348
Regularly exercise	1.008	1.003	1.013	1.027	1.001	1.054	1.023	0.999	1.047	1.006	0.998	1.013
Consume alcohol	1.221	1.118	1.334	1.123	0.648	1.944	1.119	0.708	1.768	0.971	0.844	1.117
Mental health status (MCS score)	0.998	0.993	1.002	0.983	0.963	1.004	0.996	0.978	1.015	0.991	0.984	0.997
Physical health status (PCS score)	0.981	0.977	0.985	0.986	0.964	1.007	0.974	0.956	0.992	0.985	0.978	0.991
Former smoker	1.12	1.018	1.232	2.04	1.088	3.824	1.285	0.763	2.164	1.043	0.894	1.217
Current smoker	0.737	0.65	0.836	2.759	1.455	5.233	2.05	1.207	3.48	0.853	0.705	1.033
Pregnant	1.309	0.977	1.753	–	–	–	–	–	–	–	–	–
Immunocompromised	3.768	2.532	5.607	–	–	–	–	–	–	5.475	3.607	8.31
HIV	–	–	–	–	–	–	3.192	1.841	5.535	–	–	–
Men who have sex with men	–	–	–	0.357	0.077	1.647	0.52	0.25	1.079	–	–	–
Diabetes	1.418	1.283	1.567	–	–	–	–	–	–	1.357	1.17	1.575
Asthma	1.343	1.184	1.523	–	–	–	–	–	–	–	–	–
CHD	1.133	1.004	1.28	–	–	–	–	–	–	1.397	1.179	1.656
COPD or asthma	–	–	–	–	–	–	–	–	–	1.556	1.33	1.819
COPD	1.224	1.067	1.404	–	–	–	–	–	–	–	–	–
Alcohol dependency	1.008	0.866	1.173	–	–	–	–	–	–	0.932	0.713	1.218
Liver disease	1.3	1.029	1.644	1.166	0.264	5.152	1.569	0.8	3.076	1.142	0.796	1.638
Renal disease	1.706	1.288	2.26	–	–	–	1.395	0.641	3.038	1.213	0.851	1.728

Being male and a current or former smoker were the strongest predictors of hepatitis A vaccination (see Table 3). Neither possession of health insurance, education, income, nor ethnicity was associated with greater vaccination rates. A similar pattern was observed with respect to hepatitis B vaccination in that being diagnosed with HIV, male and a current smoker were among the strongest predictors. Contrary to hepatitis A, both non-Hispanic blacks and Hispanics were significantly more likely to receive the vaccine among those at high risk.

The strongest predictors of pneumococcal vaccination were being immunocompromised, possessing health insurance, and being diagnosed with COPD or asthma, CHD, or diabetes (see Table 3). Aside from health insurance, no other sociodemographic factors were associated with vaccination behavior among those at high-risk.

## Discussion

The aim of the current study was to assess self-reported vaccination rates for different vaccines as well as to assess vaccination rates and predictors of vaccination among high-risk groups using the NHWS database. Although prior research has investigated some of these vaccines in isolation, no study had simultaneously examined several vaccines using a single database among both the general population and high-risk groups.

The data shows that influenza vaccination rates have steadily increased over the past several years. This is consistent with previous research, which found increasing vaccination rates over the past two decades, aside from years with vaccine shortages [2]–[3]. The implementation of the universal vaccination recommendation for the 2010–2011 influenza season, however did little to affect the observed trend in vaccination rates in this study. Despite the observed increase, overall rates of influenza vaccination were still low and below national targets. Approximately one-third of the adult U.S. population received the influenza vaccine in the past year whereas 100% of respondents (minus the small number with contraindications) should be have been vaccinated based on current guidelines.

The rates of vaccination for influenza among high-risk groups varied between 36.8% (pregnant women) and 69.7% (those with renal/kidney disease). Although not all of these high-risk groups have been reported in literature recently, rates among those 65 years of age and older, for example, were generally consistent between the current study (65.5%) and those in prior studies (70% in Lu et al. [2] and 65% in Lu et al. [3]). These high-risk patients are more likely to develop complications from the viral infection, yet rates of vaccination are not optimal (well below the 100% target). For other vaccines, rarely were rates of vaccination for those of high risk above 50% and, in many cases, rates were similar to those without any risk factors. For instance, men who have sex with men were less than 10% more likely to receive the hepatitis A and hepatitis B vaccine than adults without any risk factors. Those with renal/kidney disease were also only 15% more likely to receive the hepatitis B vaccine than adults without any risk factors. Compared to the general adult population, respondents with self-identified alcohol dependency were 13% more likely to receive the pneumococcal vaccine, than those without risk factors. Although direct comparisons with existing studies of hepatitis B vaccination rates are difficult because of different inclusion/exclusion criteria (e.g., the CDC only examined ages18–49), similar conclusions were reached in past studies in that only slightly higher rates were observed when comparing all adults aged 18–49 (34.6%) to those of high risk aged 18–49 (45.4%) [7]. Current smokers, despite their high-risk status, were actually less likely to receive the pneumococcal vaccine than the general population. Of course, it should be mentioned that this recommendation was made in 2008 [1] and may take additional time to be implemented.

As the number of risk factors increased, so did the probability of vaccination. However, even when looking at respondents with 5 or more risk factors for influenza complications, less than 80% reported receiving the influenza vaccine. Less than 60% of respondents with 5 or more risk factors reported receiving the pneumococcal vaccine. Although greater emphasis on vaccination for all high-risk groups is needed, it is particularly important for groups with multiple high-risk conditions. Information on contraindications was not available, so it is not known with certainty what the ideal vaccination rate should be in these cases. However, prior research has estimated the prevalence of medical exemptions for the influenza vaccine at 1.24% [16]. Contraindications vary by vaccine as does the prevalence of those contraindications, so it is difficult to generalize. Nevertheless, this does suggests that a high vaccination rate should be expected among high-risk groups if there was perfect adherence to established guidelines.

Among those at high risk, several vaccination predictors were identified. One of the strongest predictors for vaccination was having HIV or being otherwise immunocompromised. Although not relevant for hepatitis A, this does highlight the relative focus (by clinicians and/or the patients themselves) on vaccination for these patients. Few other conditions were associated as strongly with vaccination among the high-risk groups, though diabetes (for both influenza and pneumococcal) was also a relevant predictor. Insurance was a significant predictor of both influenza and pneumococcal vaccination but it was not significantly related to either hepatitis A or hepatitis B vaccination. This suggests that lack of access may be an important barrier for influenza and pneumococcal vaccination, though less so for hepatitis A and B vaccination. Ethnicity disparities were also observed in that non-Hispanic blacks and those of other ethnicities at high risk were significantly less likely to receive the influenza vaccine. Conversely, non-Hispanic blacks and Hispanics at high-risk for hepatitis B were significantly more likely to receive the vaccine. It should be emphasized that these effects were present after controlling for high-risk conditions and other sociodemographics. These ethnic differences might be the result of general healthcare and vaccination attitudes. In the case of hepatitis B, it is also possible that these discrepancies are a function of perceived medical risk for these illnesses.

Although the focus on this paper was the United States, it is worth making some broad international comparisons for a global perspective on these issues. For example, the CDC has issued a universal recommendation for the influenza vaccine, yet most other nations recommend only on a subset of the population to receive the influenza vaccination [17]. This difference in policy may result in lower rates of vaccination overall and among high risk groups in these countries compared with the United States. Indeed, a recent study using a similar methodology has reported influenza vaccination rates in Europe lower than in the present analysis [18].

In summary, these results suggest that vaccination rates remain suboptimal, even among high-risk groups for many vaccines. Rates for the influenza vaccination have shown a consistent increase over the past several years. Although only assessed for influenza vaccines, reasons for not getting vaccinated suggest the major obstacle is overcoming the belief that the vaccine is either unimportant or ineffective. Greater clinical emphasis on the importance of vaccination, particularly for minorities and the high-risk populations for these vaccine-preventable diseases may help address this important public health issue. Of course, it is also important to emphasize that the differences in the vaccine regimen (e.g., annual vaccine versus one-time vaccine) are important to consider and strategies to increase vaccination rates must be tailored to the specific vaccine in question.

### Limitations

The NHWS is a self-reported health survey and no verification of vaccine receipt was available. Therefore, the sensitivity and specificity of the self-report vaccine questions remains unknown. Recall biases, particularly when asking respondents if they have ever received a particular vaccine, may have introduced additional error into the estimated vaccination rates. Although some vaccination (influenza, pneumococcal for high-risk patients 19–64, hepatitis B) rates are generally similar to what is reported by the NHIS, rates of Td, Tdap, herpes zoster, hepatitis A, and pneumococcal for patients 65 and older were substantially different in the current study (see Appendix 2). This may have introduced additional error. Our approach to separate out the combination vaccine into both receiving hepatitis A and hepatitis B was done to minimize measurement error (so patients would not need to recall whether their hepatitis A vaccine they received was received in isolation or as part of a combination). Although we felt this was the best approach given the NHWS data, it does prevent a complete understanding of differences between patients who receive hepatitis vaccines in isolation or as part of a combination. Future research is necessary.

Although an attempt was made to match CDC high-risk group definitions as closely as possible, not all high-risk subgroups could be defined with NHWS data, and proxies may not exactly parallel CDC recommendations. For example, those who are traveling to certain countries, are injection drug users, or have certain sexually transmitted diseases are recommended to receive the hepatitis B vaccine. Additionally, vaccination for hepatitis A is recommended for adults with certain risks related to their health, job, or lifestyle, which may not be captured in the survey. This remains a limitation. The number of respondents with additional risk factors and the accuracy of reporting risk-increasing behavior are unknown. However, most risk factors were medical conditions and prior studies have shown congruence between comorbidities assessed in the NHWS and comorbidities assessed in other governmental sources such as National Health and Nutrition Examination Survey (NHANES), NHIS, and Medical Expenditure Panel Survey (MEPS) [19]–[21]. Although this does not assure each risk factor was accurate for each patient, it does suggest a limited (and randomly-distributed) amount of error in the reporting these risk factors.

The NHWS uses a stratified random sample to mimic the age, gender, and racial/ethnic composition of the U.S. adult population, the source of the sample was an Internet panel and those who chose to respond to the NHWS may differ in meaningful ways other than demographics. As a result, it is unclear the extent to which these results generalize to the total adult population. Also relevant to the discussion of the external validity of the results is the fact that local differences in the uptake of immunizations and regulations for school-enrollment and occupations that may influence vaccine uptake. This suggests that the results in the current study may not generalize to all specific subpopulations in the US, as there may be variability in vaccination rates.

## Supporting Information

Table S1 **Demographic comparison of NHWS respondents and the Current Population Survey of the U.S. Census.**
(DOCX)

Table S2 **Comparisons of vaccination rate estimates between the National Health Interview Survey and the National Health and Wellness Survey.**
(DOCX)

## References

[1] Centers for Disease Control and Prevention (CDC) (2011) Recommended adult immunization schedule – United States, 2011. MMWR 60: 1–4. 21381442

[2] Lu P, Bridges CB, Euler GL, Singleton JA (2008) Influenza vaccination of recommended adult populations, U.S., 1989–2005. Vaccine 26: 1786–1793. 18336965 10.1016/j.vaccine.2008.01.040

[3] Lu P, Singleton JA, Rangel MC, Wortley PM, Bridges CB (2005) Influenza vaccination trends among adults 65 years or older in the United States, 1989–2002. Arch Intern Med 165: 1849–1856. 16157828 10.1001/archinte.165.16.1849

[4] Centers for Disease Control and Prevention (CDC) (2005) Influenza vaccination levels among persons aged >65 years and among persons aged 18–64 years with high-risk conditions – United States, 2003. MMWR 54: 1045–1049. 16237375

[5] Egede LE, Zheng D (2003) Racial/ethnic differences in influenza vaccination coverage in high-risk adults. Am J Public Health 93: 2074–2078. 14652337 10.2105/ajph.93.12.2074 PMC1448155

[6] Centers for Disease Control and Prevention (CDC) (2008) Influenza vaccination coverage among persons with asthma – United States, 2005–06 influenza season. MMWR 57: 653–657. 18566564

[7] Singleton JA, Wortley P, Lu P (2004) Influenza vaccination of persons with cardiovascular disease in the United States. Tex Heart Inst J 31: 22–27. 15061622 PMC387428

[8] Jain N, Yusuf H, Wortley PM, Euler GL, Walton S, Stokley S (2004) Factors associated with receiving hepatitis B vaccination among high-risk adults in the United States: An analysis of the National Health Interview Survey, 2000. Fam Med 36: 480–486. 15243828

[9] Centers for Disease Control and Prevention (CDC) (2006) Hepatitis B vaccination coverage among adults – United States, 2004. MMWR 55: 509–511. 16691181

[10] Lu PJ, Byrd KK, Murphy TV, Weinbaum C (2011) Hepatitis B vaccination coverage among high-risk adults 18–49 years, U.S., 2009. Vaccine 29: 7049–7057. 21782873 10.1016/j.vaccine.2011.07.030

[11] Lightspeed Online Research, Inc website. Available: http://www.lightspeedresearch.com/online-market-research-survey-panels. Accessed 2011 Oct 26.

[12] United States Bureau of Census for the Bureau of Labor Statistics, Current Population Survey website. Available: http://www.bls.census.gov/cps_ftp.html. Accessed 2011 Oct 26.

[13] Horvitz DG, Thompson DJ (1952) A generalization of sampling without replacement from a finite universe. J Am Stat Assoc 47: 663–685.

[14] Ware JE, Kosinski M, Turner-Bowker DM, Gandek B (2002) How to Score the Version 2 of the SF-12 Health Survey (with a supplement documenting version 1). Lincoln, RI: Quality Metric.

[15] Centers for Disease Control and Prevention. Shingles vaccination: What you need to know. Available: http://www.cdc.gov/vaccines/vpd-vac/shingles/vacc-need-know.htm. Accessed 2012 Jan 11.

[16] Babcock HM, Gemeinhart N, Jones M, Dunagan WC, Woeltje KF (2010) Mandatory influenza vaccination of health care workers: Translating policy to practice. Clin Infect Dis 50: 459–464. 20064039 10.1086/650752

[17] World Health Organization. Influenza vaccines: Position paper (2005) Weekly Epid Record. 33: 279–287.

[18] DiBonaventura MD, Goren A, Gupta S, Wagner JS, Freedman D (2010) Influenza risk and vaccination rates in Europe: A nationwide survey of adults Presentation at the International Society of Pharmacoeconomics and Outcomes Research 13^th^ Annual European Conference. Prague, Czech Republic. November 2010.

[19] DiBonaventura MD, Wagner JS, Yuan Y, L’Italien G, Langley P, Ray Kim W (2010) Humanistic and economic impacts of hepatitis C infection in the United States. J Med Econ 13: 709–718. 21091098 10.3111/13696998.2010.535576

[20] Finkelstein EA, Allaire BT, DiBonaventura MD, Burgess SM (2011) Direct and indirect costs and potential cost savings of laparoscopic adjustable gastric banding among obese patients with diabetes. J Occup Environ Med 53: 1025–1029. 21866052 10.1097/JOM.0b013e318229aae4

[21] Bolge SC, Doan JF, Kannan H, Baran RW (2009) Association of insomnia with quality of life, work productivity, and activity impairment. Quality of Life Research 18: 415–422. 19288223 10.1007/s11136-009-9462-6

