# Supplementary material for: Vaccination Rates among the General Adult Population and High-Risk Groups in the United States
Source: PLoS One. 2012 Nov 30;7(11):e50553. doi: 10.1371/journal.pone.0050553 (PMC3511565; doi:10.1371/journal.pone.0050553)
Supplement: Table S1 — Demographic comparison of NHWS respondents and the Current Population Survey of the U.S. Census. (DOCX) [file pone.0050553.s001.docx]

|  | | **NHWS 2010 (Unweighted)** | **NHWS 2010 (Weighted)** | **Current Population Survey 2009 (U.S. Census)** |
| --- | --- | --- | --- | --- |
| **Gender** | **Male** | 48.2% | 48.3% | 48.3% |
|  | **Female** | 51.8% | 51.7% | 51.7% |
| **Age** | **18-44** | 43.5% | 48.6% | 49.1% |
|  | **45-64** | 34.5% | 34.7% | 34.4% |
|  | **65+** | 22.1% | 16.7% | 16.5% |
| **Race** | **African-American** | 10.7% | 11.4% | 11.3% |
|  | **Hispanic** | 6.4% | 13.5% | 13.5% |
|  | **White** | 74.1% | 68.5% | 68.8% |
|  | **Other** | 8.8% | 6.6% | 6.4% |
| **Income** | **< $25,000** | 18.2% | 19.1% | 18.3% |
|  | **$25,000-$49,999** | 26.9% | 27.3% | 22.3% |
|  | **$50,000-$74,999** | 19.7% | 19.7% | 16.8% |
|  | **$75,000 or more** | 27.0% | 26.1% | 25.6% |
| **Education** | **Some college or less** | 58.9% | 60.3% | 73.0% |
|  | **College graduate** | 41.1% | 39.7% | 27.1% |
| **Region** | **Northeast** | 18.8% | 18.6% | 18.5% |
|  | **Midwest** | 23.9% | 23.1% | 22.1% |
|  | **South** | 35.4% | 35.8% | 36.4% |
|  | **West** | 22.0% | 22.5% | 23.1% |
