# Supplementary material for: Vaccination Rates among the General Adult Population and High-Risk Groups in the United States
Source: PLoS One. 2012 Nov 30;7(11):e50553. doi: 10.1371/journal.pone.0050553 (PMC3511565; doi:10.1371/journal.pone.0050553)
Supplement: Table S2 — Comparisons of vaccination rate estimates between the National Health Interview Survey and the National Health and Wellness Survey. (DOCX) [file pone.0050553.s002.docx]

|  |  | **National Health Interview Survey (2009)** | **National Health and Wellness Survey comparisons (2011)** |
| --- | --- | --- | --- |
| *Influenza (received in the past year)* | |  |  |
|  | 19 years and over, total | 33.6% | 36.3% |
|  | 50-64 years, total | 40.1% | 42.0% |
|  | 65 years and over, total | 65.6% | 65.5% |
|  |  |  |  |
| *Pneumonoccocal vaccine (ever received)* | |  |  |
|  | 19-64 years, high-risk, total | 17.5% | 20.2% |
|  | 65 years and over, total | 60.6% | 50.4% |
|  |  |  |  |
| *Hepatitis A vaccine (at least 2 doses)* | |  |  |
|  | 19-49 years, high-risk, total | 9.8% | 26.1% |
|  |  |  |  |
| *Hepatitis B vaccine (at least 3 doses)* | |  |  |
|  | 19-49 years, high-risk, total | 41.8% | 39.5% |
|  | 19-49 years, non-high-risk total | 33.7% | 32.3% |
|  |  |  |  |
| *Tdap vaccine (received in the past year)* | |  |  |
|  | 19-64 years, total | 6.6% | 4.6% |
| *Herpes zoster (ever received)* | |  |  |
|  | 60 years and over, total | 10.0% | 7.9%* |
|  | |  |  |
|  |  |  |  |

**Estimated from the 2010 NHWS.*

*NHIS data estimated that 15.5% of those aged 19-64 received the Td vaccine in the past five years while NHWS data estimated that 28.1% of those aged 19-64 had ever received the vaccine.*

*NHIS data estimated that 61.3% of those aged 19 and older had received the tetanus vaccine in the past 10 years while NHWS data estimated that 42.1% had ever received the Td vaccine.*
